# Supplementary material for: Transcriptome Analysis of the Influence of High-Pressure Carbon Dioxide on Saccharomyces cerevisiae under Sub-Lethal Condition
Source: J Fungi (Basel). 2022 Sep 27;8(10):1011. doi: 10.3390/jof8101011 (PMC9605315; doi:10.3390/jof8101011)
Supplement: Supplementary file 1 [file jof-08-01011-s001.zip › jof-1886112-supplementary.pdf]

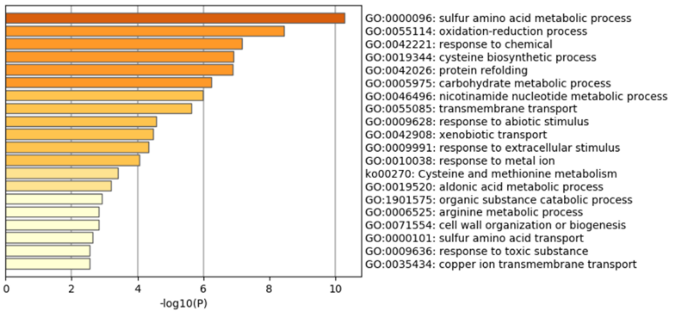

A

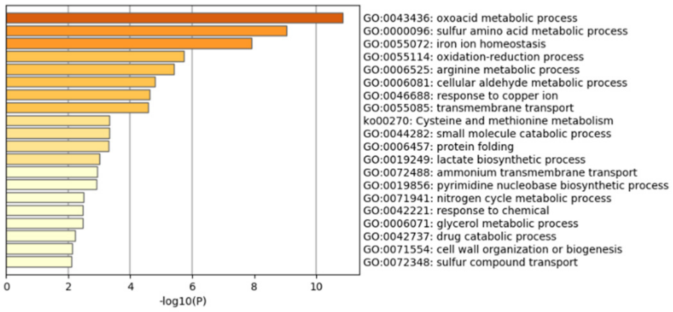

B

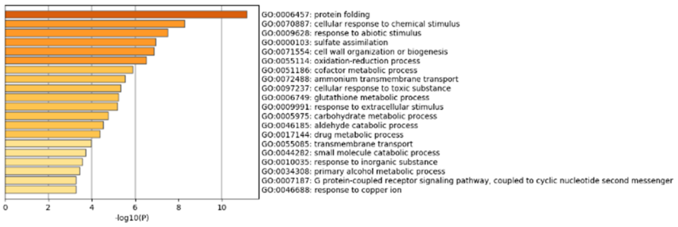

C

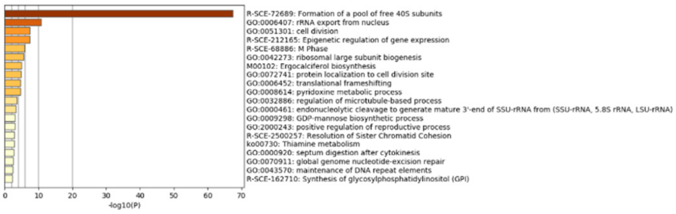

D

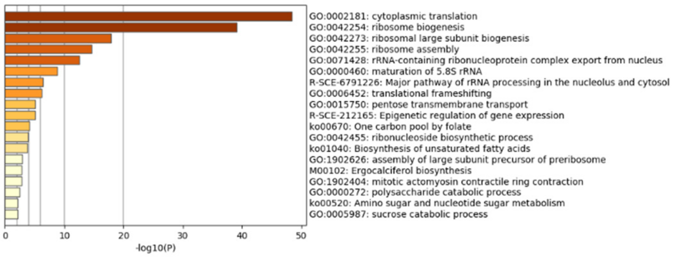

E

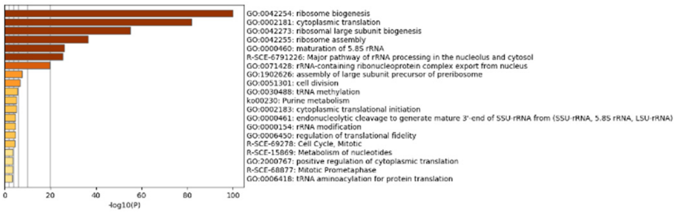

F

Figure S1. Gene ontology (GO) analysis of the identified DEGs. A and D are after 1-h treatment, B and E are after 2 h of treatment, and C and F are after 4 h of treatment. A to C indicate up-regulated genes, and D to F indicate down-regulated genes.
